# Editorial: Rising stars in data mining and management 2022

**DOI:** 10.3389/fdata.2023.1201798

**Published:** 2023-06-22

**Authors:** Feng Chen, Shuhan Yuan, Jilin Hu, Cheng-Te Li, Rudy Raymond, Lidan Shou

**Affiliations:** ^1^Department of Computer Science, The University of Texas at Dallas, Richardson, TX, United States; ^2^Department of Computer Science, Utah State University, Logan, UT, United States; ^3^Department of Computer Science, Aalborg University, Aalborg, Denmark; ^4^Institute of Data Science and Department of Statistics, National Cheng Kung University, Tainan, Taiwan; ^5^IBM Japan, Tokyo, Japan; ^6^College of Computer Science and Technology, Zhejiang University, Hangzhou, China

**Keywords:** data mining, knowledge graph, autonomous sensor configuration, transfer learning, graph mining, spatial data

This Research Topic is to exhibit the high-quality work of rising stars, promising researchers who have gained international reputations in the early stages of research career. The aim of developing this Research Topic is to encourage researchers to attend to and further explore these emerging territories of Data Mining and Management. We showcase the research in several representative areas of Data Mining and Management conducted by these rising star scientists. The topic includes five papers to appear in Frontiers in Big Data, including four original research articles and one review article. The articles cover a range of areas, including spatial data analysis, knowledge-infused learning, transfer learning, and graph mining.

The article by Li et al. (“*Spatial data analysis for intelligent buildings: awareness of context and data uncertainty*”) sheds light on two critical challenges in spatial data analysis for intelligent buildings. The first challenge is the complex analytical contexts of building spaces and internal entities, while the second challenge is the high spatial data uncertainty due to limitations in positioning and sensing technologies. The authors present recent advancements in addressing these challenges and propose a framework to accommodate modeling techniques for various analytical contexts and spatial data uncertainties. Moreover, the authors explore emerging opportunities and ongoing issues in the new technology ecosystem.

The article by Rohal et al. (“*AutoLoc: Autonomous sensor location configuration via cross modal sensing*”) delves into the issue of autonomous localization in floor vibration-based occupant monitoring systems. The article focuses on the applications of this technology in non-intrusive in-home continuous occupant monitoring. The authors identify three primary challenges: accurate footstep event prediction and localization via video signal with noisy posture estimation, solving multilateration equations with unknown vibration propagation velocity, and footstep event selection from accumulating sensor data to achieve precise vibration sensor localization. The authors propose a scheme combining physical and data-driven knowledge to overcome these challenges.

Knowledge-infused learning has attracted tremendous attention thanks to the improved knowledge graphs that can represent meaningful relations between entities at a large scale. The paper entitled “*Knowledge-infused learning for entity prediction in driving scenes*” by Wickramarachchi et al. addresses the challenge of scene understanding within the autonomous driving domain. The authors leverage heterogeneous, high-level semantic knowledge graphs of driving scenes to predict potentially unrecognized entities and improve driving-scene understanding. They developed an innovative neuro-symbolic solution that utilizes an expressive, holistic representation of the scene with knowledge graphs and conducts entity prediction based on knowledge-graph embeddings.

In the era of big data, graph structures are widely used to represent complex relational information. Therefore, various graph-based algorithms and tools have been developed to address different real-world tasks. The article by Fu and He (“*Natural and artificial dynamics in graphs: concept, progress, and future*”) provides a comprehensive review of graph-based algorithms and tools with a focus on the topic of natural dynamics and artificial dynamics in graphs. Natural dynamics refers to the evolving topology structures, node-level, edge-level, and subgraph-level features and labels of input graphs, while artificial dynamics involves changes made by end-users to existing or non-existing graph-related elements to improve performance. In this article, the authors first introduce three topics in graph research, namely graph mining, graph representations, and graph neural networks. Then, the authors present the definitions of natural dynamics and artificial dynamics in graphs and their related studies as well as discuss the interplay between natural and artificial dynamics and their impact on graph Research Topics.

Dynamic transfer learning indicates transferring knowledge from a static source task that has sufficient label information to a dynamic target task hat lacks label information. Although most current theoretical studies assume that the target task evolves continuously over time, this is not always the case in real-world scenarios, where the target distribution may suddenly change. To address this limitation, the article by Wu and He (“*Dynamic transfer learning with progressive meta-task scheduler*”) develops a meta-learning framework, denoted as L2S, for dynamic transfer learning based on a progressive meta-task scheduler. L2S learns to schedule meta-pairs of tasks and uses them to determine the optimal model initialization for rapid adaptation. The article demonstrates the effectiveness through theoretical and empirical studies.

The articles included in this Research Topic exhibit only part of the studies contributed by the rising stars. Interested readers are encouraged to follow their ongoing work to pursue the research frontier of the relevant areas. We extend our gratitude to the authors and referees for their significant contributions and efforts toward making this Research Topic possible.

## Author contributions

SY and FC drafted the manuscript. All authors contributed to the article and approved the submitted version.

